# 4134 Report from the research trenches: A mixed-methods approach to investigation of how recruitment methods, culture and collaboration impact clinical trial accrual

**DOI:** 10.1017/cts.2020.242

**Published:** 2020-07-29

**Authors:** Kitt Swartz, Meredith Zauflik, Adrienne zell, Cynthia Morris, David Ellison

**Affiliations:** 1 Oregon Health & Science University

## Abstract

OBJECTIVES/GOALS: The research project aimed to understand the perceived effectiveness of research recruitment methods, including informatics tool utilization, so that best practices can be established and outcomes measured longitudinally. METHODS/STUDY POPULATION: The mixed-methods study was conducted by the Oregon Clinical and Translational Science Institute, the CTSA at Oregon Health and Sciences University. A survey, clinical trial accrual data, and interviews were used to assess the study aims. The survey asked about utilization and value of specific recruitment tools and methods. Accrual data was obtained from the clinical trial management system and analyzed using parameters from the CTSA “Accrual Metric”. The metric was calculated for clinical trials enrolling during 2017. Interviews were conducted with researchers identified by the survey and over or under-enrolled accrual data, and inquired about recruitment facilitators and barriers. RESULTS/ANTICIPATED RESULTS: The most frequently mentioned facilitator of recruitment was direct patient contact, either in the healthcare setting (58.4% of survey respondents) or through patient outreach (32%). A lack of resources was considered a key barrier (21% of survey respondents) and a stated need (27%). Interview data expanded on these findings, as 23% of interviewees indicated a collaborative culture, which includes clinic integration, was key to recruitment success. Additionally, 20% of interviewees identified resources (i.e. funding, staff, time) as their greatest need. Notably, 13% of studies with an accrual ratio of “0” had frequent staff turnover. DISCUSSION/SIGNIFICANCE OF IMPACT: This approach allowed for a uniquely targeted analysis of accrual facilitators and barriers. Use of the CTSA accrual metric identified high-value interview respondents and will allow for investigation into additional accrual questions, such as the impact of funding sources and departmental factors.

